# Ubiquitin Specific Protease 21 Is Dispensable for Normal Development, Hematopoiesis and Lymphocyte Differentiation

**DOI:** 10.1371/journal.pone.0117304

**Published:** 2015-02-13

**Authors:** Jaspreet Pannu, Jad I. Belle, Michael Förster, Claudia U. Duerr, Shiyang Shen, Leanne Kane, Katherine Harcourt, Jörg H. Fritz, Simon Clare, Anastasia Nijnik

**Affiliations:** 1 Department of Physiology, McGill University, Montreal, Canada; 2 Department of Biology, McGill University, Montreal, Canada; 3 Complex Traits Group, McGill University, Montreal, Canada; 4 Department of Microbiology and Immunology, McGill University, Montreal, Canada; 5 Wellcome Trust Sanger Institute, Wellcome Trust Genome Campus, Hinxton, United Kingdom; Northern Institute for Cancer Research, UNITED KINGDOM

## Abstract

USP21 is a ubiquitin specific protease that catalyzes protein deubiquitination, however the identification of its physiological substrates remains challenging. USP21 is known to deubiquitinate transcription factor GATA3 and death-domain kinase RIPK1 *in vitro*, however the *in vivo* settings where this regulation plays a biologically significant role remain unknown. In order to determine whether USP21 is an essential and non-redundant regulator of GATA3 or RIPK1 activity *in vivo*, we characterized *Usp21*-deficient mice, focusing on mouse viability and development, hematopoietic stem cell function, and lymphocyte differentiation. The *Usp21*-knockout mice were found to be viable and fertile, with no significant dysmorphology, in contrast to the GATA3 and RIPK1 knockout lines that exhibit embryonic or perinatal lethality. Loss of USP21 also had no effect on hematopoietic stem cell function, lymphocyte development, or the responses of antigen presenting cells to TLR and TNFR stimulation. GATA3 levels in hematopoietic stem cells or T lymphocytes remained unchanged. We observed that aged *Usp21*-knockout mice exhibited spontaneous T cell activation, however this was not linked to altered GATA3 levels in the affected cells. The contrast in the phenotype of the *Usp21*-knockout line with the previously characterized GATA3 and RIPK1 knockout mice strongly indicates that USP21 is redundant for the regulation of GATA3 and RIPK1 activity during mouse development, in hematopoietic stem cells, and in lymphocyte differentiation. The *Usp21*-deficient mouse line characterized in this study may serve as a useful tool for the future characterization of USP21 physiological functions.

## Introduction

USP21 is a member of the ubiquitin specific protease (USP) family of deubiquitinating enzymes (DUBs) [1,2]. It can catalyze the proteolysis of a variety of ubiquitin chains, including K6-, K11-, K29-, K48-, K63- and linear ubiquitin conjugates [3]. It is also reported to degrade conjugates of ubiquitin-like protein ISG15 [3], and according to some reports NEDD8 [4].

Identification of the physiological protein substrates that are deubiquitinated by USP21 remains challenging. USP21 was originally demonstrated to remove ubiquitin from histone H2A (H2AK119ub), with strong specificity for nucleosomal rather than free histone substrates [5]. This activity was suggested to promote transcriptional activation, especially in mouse hepatocytes during liver regeneration [5]. Subsequently two splice isoforms of USP21 protein were identified [6], and the short isoform was shown to localize primarily to the nucleus and the nuclear membrane, and to function as a deubiquitinase for histone H2A [6]. In contrast the long isoform of USP21 was shown to localize to the cytosol and associate with microtubules [7,8]. Several other substrates of USP21 were identified, namely transcription factor GATA3, death-domain containing protein kinase RIPK1 [9,10], and most recently the anti-viral pattern recognition receptor RIG-I and cytokine IL-33 [11,12]. Specifically, USP21 was shown to deubiquitinate and stabilize GATA3 *in vitro* and in cell lines, promoting GATA3 transcriptional activity [10]. In contrast, USP21 acted as a negative regulator of RIPK1, inhibiting its activity downstream of TNFα receptor 1 (TNFR1), and therefore suppressing signaling through the NFκB pathway and inflammatory cytokine production [9].

GATA3 is a zinc finger transcription factor essential for normal embryonic development in mouse models, and GATA3-deficient mouse embryos die at day 11.5 dpc with severe abnormalities in the nervous system, kidneys, vasculature, and fetal hematopoiesis [13]. GATA3 is also essential for hematopoietic and immune systems in the adult mouse. Thus GATA3 regulates cell cycle progression and self-renewal capacity of bone marrow hematopoietic stem cells (HSCs) [14,15]. GATA3 also plays a critical role at all stages of T cell development in the thymus [16,17], and in the functions of Th2-polarized effector T cells and regulatory T cells (Tregs) [18,19,20].

RIPK1 is a serine/threonine kinase with a C-terminal death-domain. It is a critical component of the signaling pathways mediating NFκB activation, inflammatory cytokine production, and cell death through necroptosis, downstream of receptors TNFR1, TLR3, TLR4, TRAIL-R1/R2, Fas and others [21,22,23]. RIPK1 activity is regulated through ubiquitination, including K63-polyubiquitination that mediates downstream NFκB activation, and K48-polyubiquitination that targets RIPK1 for proteasome-dependent degradation [24,25]. Stringent regulation of RIPK1 activity is essential for mammalian development, viability, and immune functions. RIPK1-deficient cells are hyper-susceptible to TNFα-induced apoptosis, and RIPK1-knockout mice die at days 1–3 after birth, due high levels of cell death in many tissues, including immune and lymphoid organs [26,27]. Similarly, increased RIPK1 levels and activity, which occur for example in a FADD-deficient mouse line, result in lethality with elevated levels of necrosis, as well as impaired T cell proliferation [28,29].

USP21 is also known to hydrolyze conjugates of the ubiquitin-like protein ISG15 [3], however the specific protein substrates the ISG15-conjugation state of which is modulated by USP21 have not been identified. Importantly, ISG15 is strongly induced in response to type-I interferons (IFNs), and ISG15-conjugation of multiple protein substrates plays an essential role in the mammalian immune responses to infections [30,31,32]. For example, ISG15-deficiency in humans is linked with increased susceptibility to Mycobacterial infections [32], while deficiency in ISG15-specific protease Ubp43 in mouse models results in increased resistance to *S. typhimurium* challenge [30]. The possible roles of USP21 in immune responses to bacterial infection currently remain untested.

In summary, although USP21 was shown to modulate the ubiquitination state, stability and activity of GATA3 and RIPK1 *in vitro* and in cell lines [9,10], the *in vivo* settings where this regulation plays a biologically significant role remain unknown. Here we characterize a *Usp21*-deficient mouse line and observe no gross abnormalities in overall morphology, hematopoietic stem cell maintenance, lymphocyte differentiation, or resistance to bacterial challenge, suggesting that USP21 is redundant as a regulator of GATA3 or RIPK1 functions in these settings.

## Materials and Methods

### Mouse Line

The mouse line carrying the *Usp21* ^tm1a(EUCOMM)Wtsi^ allele was generated by blastocyst injection of JM8A3.N1 embryonic stem cell (ESC) clone EPD0716_10_H09, generated by the International Mouse Knockout Consortium [33,34]. The identity of the targeted ESCs was verified by long-range PCR using a primer external to the targeting vector. Chimeric mice were bred to C57BL/6-Tyrc-Brd and germline transmission was verified in F1 heterozygous mice by quantitative-PCR (qPCR), to detect the copy numbers of the neo transgene and the wild-type allele, confirming single insertion event and correct targeted locus, respectively. The presence of the downstream loxP site was verified by PCR. The study was approved by the McGill University Animal Care Committee. The care and use of all mice was in accordance with the guidelines of Canadian Council on Animal Care, or the UK Home Office regulations, UK Animals Scientific Procedures Act 1986. The mouse line is available for distribution as frozen sperm from EMMA: https://www.infrafrontier.eu/search?keyword=EM:07280.

### Mouse Genotyping

Mice were genotyped using DNA prepared from earclip tissue, using Platinum Taq Polymerase (Life Technologies) with the following primers: USP21_WT_FW AGTACTTCTCTCCGGCGTCTTG, USP21_WT_RV AGAGAGTGCCTAAGGACCCAAG, CAS_Rv_USP21 TCATCAGAAGCAGGCCACCCAA.

### RNA isolation and qRT-PCR

RNA extraction was performed using either Trizol (Invitrogen, Life Technologies) or EZ-10 DNAaway RNA Miniprep Kit (BioBasic Inc.) according to the manufacturers’ protocols. RNA integrity was assessed by spectrophotometry and agarose gel electrophoresis. First strand cDNA synthesis was performed with the M-MLV Reverse Transcriptase kit (Invitrogen, Life Technologies) utilizing random hexamer primers (Invitrogen, Life Technologies). Real-time qPCR analysis was done on an Applied Biosystems StepOnePlus instrument with Power SYBR Master Mix (Applied Biosystems, Life Technologies). *Usp21* primers were Usp21_Fw CTGAGCCTTTCTACTCTGATGAC, Usp21_Rv GGCATTCAGGAAGCATGTATTT. Additional primer sequences for *Usp1, Usp2, Usp12, Usp43, Usp46, Mapk2*, and housekeeping control genes are provided in S1 Table

### Tissue and Blood Collection

Spleen, bone marrow, thymus and mesenteric lymph nodes were collected and processed to single-cell suspension in RPMI-1640 (Wisent) with 10% (v/v) Fetal Calf Serum (FCS), 2mM L-glutamine, 1mM sodium pyruvate, 100 μg/ml streptomycin, 100 U/ml penicillin, and 1μg/ml Amphotericin B (Wisent). To collect blood from live mice, submandibular bleeds were performed into tubes containing 10mM EDTA in PBS to prevent clotting. Erythrocytes were lysed in Tris-NH_4_Cl (17mM Tris Base, 140mM NH_4_Cl, pH7.2) at 37°C, three incubations of 10 minutes.

### Mouse bone marrow transfer experiments

For bone marrow chimeras experiment, recipient mice of the C57BL/6J or B6.SJL-Ptprca Pepcb/Boy (JAX 002014) strains were irradiated with 2 doses of 4.5Gy, delivered 3 hours apart, in the RS2000 irradiator (Rad Source Technologies). The mice were then intravenously injected with 2.5 x 10^6^ bone marrow cells from B6.SJL-Ptprca and *Usp21* ^-/-^ donor mice, mixed in a 1:1 ratio. The chimeric mice were kept on neomycin in drinking water (2 g/l, BioShop) for 3 week, bled at 8 weeks and sacked for full analysis at 20 weeks after the reconstitution. The relative contribution of *Usp21* ^-/-^ donor cells to the different hematopoietic and immune cell lineages was measured, with 50% average contribution indicating normal *Usp21* ^-/-^ hematopoietic stem cell functions and normal *Usp21* ^-/-^ contribution to hematopoiesis in direct competition with wild type cells.

### Infection of mice

8 *Usp21*-knockout and 8 wild type control mice were infected intravenously with 5 x 10^5^ cfu *Salmonella typhimurium* M525 TETc at 6 weeks of age and monitored over 28 days. On day 14 post-infection, 4 of the mutant mice and 4 wild type control mice were killed, and their spleen and liver were extracted and bacteria enumerated by serial dilution and plating onto agar plates containing 50μg/ml ampicillin. On day 28 post-infection the remaining mice were bled under terminal anaesthesia via cardiac puncture. Organs were removed and processed in the same way as describe for day 14 post infection. Blood was centrifuged at 16 000g for 5 minutes, serum was removed and tested for anti TETc specific antibodies by ELISA assay (IgG, IgG1 and IgG2a). Mice were weighed daily throughout the infection.

### Differentiation of Macrophages and Dendritic Cells from Mouse Bone Marrow

For the differentiation of bone marrow derived macrophages (BMDMs), 4 x10^6^ mouse bone marrow cells were plated into 5 mLs of DMEM (Wisent) with 20% FCS, 2mM L-Glutamine, 1mM sodium pyruvate, 100μg/ml streptomycin, 100U/ml penicillin, 1μg/ml amphotericin B (all from Wisent), and 25% L929 cell conditioned media (prepared in house according to standard protocols, contains M-CSF). For the differentiation of bone marrow derived dendritic cells (BMDCs), 4 x10^6^ mouse bone marrow cells were plated into 5 mLs of RPMI-1640 (Wisent) with 10% FCS, 2mM L-Glutamine, 1mM pyruvate, 100μg/ml streptomycin, 100U/ml penicillin, 1μg/ml amphotericin B (all from Wisent), 50 μM β-mercaptoethanol (Calbiochem), and 25 ng/mL GM-CSF (Peprotech). The cells were maintained at 37°C and 5% CO_2_ in a humidified incubator for 1 week. To detach, the cells were washed in PBS (Wisent) and treated with Versene (0.02% w/w EDTA in PBS, Wisent). Successful derivation of macrophages and dendritic cells was confirmed by the changing morphology of the cells and by flow cytometry analysis for the expression of CD11b, F4/80 and CD11c lineage markers.

### Cell Stimulation Protocols

For the stimulation of BMDCs and BMDMs the cells were re-plated at 50,000 or 100,000 cells/well, respectively, in the total volume of 150 μL/well, in the same media as used in the cell-differentiation protocols described above. The cells were stimulated overnight with LPS at 10 ng/mL or 100 ng/mL (LPS *E.coli* O111:B4, Millipore), with poly(IC) at 5 μg/ml or 25 μg/ml (Poly(I:C) HMW, Invivogen), or with mouse recombinant TNFα at 5 ng/ml or 10 ng/ml (eBioscience). During the stimulation the cells were maintained at 37°C and 5% CO_2_ in a humidified incubator.

### Flow Cytometry

For cell-surface marker analysis the cells were stained in PBS with 2% FCS and 0.2% (w/v) sodium azide (VWR, Amresco) for 20 minutes on ice, with fluorophore-conjugated antibodies (from BD Biosciences, BioLegend, eBioscience or Tonbo Biosciences; full antibody list provided in S2 Table). For intracellular staining, the cells were fixed in 2% paraformaldehyde in PBS with 2% FCS at 37°C for 10 minutes, permeabilized in 90% methanol in PBS with 2% FCS for 30 minutes on ice, and stained for 1 hour at room temperature with Alexa Fluor 647 anti-IκBα (L35A5), PE anti-phospho-NFκB p65 Ser536 (93H1; both from Cell Signaling), Alexa Fluor 488 anti-GATA3 (16E10A23) or Alexa Fluor 488 Mouse IgG2b isotype control (both from BioLegend). Fixable Viability Dye eFluor 506 (eBioscience) was used to discriminate live and dead cells. Compensation was performed with BD CompBeads (BD Biosciences). The data were acquired on FACS Canto II flow cytometer (BD Biosciences) and analyzed with FACS Diva (BD Biosciences) or FlowJo (Tree Star) software.

For flow cytometry analysis of cytokine production the cells were treated with Brefeldin A (eBioscience) at 3 μg/ml, starting at 2 hours after the beginning of the stimulation and continuing overnight. The cells were then pre-stained for cell-surface markers CD11b, CD11c, F4/80 as described above. The cells were fixed with 2% paraformaldehyde (VWR, Amresco) in PBS with 2% FCS (PAA Laboratories) for 10 minutes at room temperature and permeabilized with 0.1% saponin in PBS with 4% FCS (PAA Laboratories) for 15 minutes on ice. The cells were then stained for 1 hour on ice with the following anti-cytokine antibodies pre-diluted in the permeabilization buffer according to manufacturer’s instructions: PE-conjugated anti-mouse IL-6 (clone MP5-20F3) and APC-conjugated anti-mouse TNFα (MP6-XT22, both from eBioscience).

### ELISA

Tissue culture supernatants were centrifuged to obtain cell-free samples, and stored at −20°C. Cytokine levels were measured by sandwich ELISA, using anti-mouse TNF-α antibody clones 1F3F3D4 and XT3/XT22 and anti-mouse IL-6 antibody clones MP5-20F3 and MP5-32C11 (all from eBioscience), followed by avidin horseradish peroxidase (eBioscience), as per manufacturer’s protocols. The ELISAs were developed using SuperAquaBlue ELISA Substrate (eBioscience) and imaged with on an EnSpire 2300 Multilabel Reader (Perkin Elmer). Throughout the ELISA procedure PBS with 0.05% Tween-20 was used as the Wash Buffer, and PBS with 1% Bovine Serum Albumin as the Blocking Buffer and Assay Diluent. Recombinant murine IL-6 (Peprotech) and TNFα (eBioscience) were used as standards for the calculation of cytokine concentrations.

For the measurements of mouse serum antibody titres, mouse blood was collected by cardiac puncture, and serum was prepared and stored at −20°C. Nunc MaxiSorp plates were coated overnight at 4°C with 2 mg/ml tetanus toxin fragment C recombinant protein (TetC) in 0.1 M Na_2_HPO_4_ (pH 9.0), blocked with 3% (w/v) BSA in PBS for 1 hour, and incubated with 5-fold serial dilutions of mouse serum in PBS with 1% BSA for 1 hour. The plates were developed with anti-mouse IgG, IgG1, or IgG2a HRP-conjugated Abs (BD Biosciences), followed by o-phenylenediamine substrate tablets (Sigma-Aldrich) dissolved in water. Absorbance was measured using the Bio-Rad 680 microplate reader.

### Statistical analyses

Statistical comparisons were performed with Prism 4.0 (GraphPad Inc.), using Mann-Whitney test for comparisons of two datasets, and ANOVA with Bonferroni post-hoc test for multiple comparisons.

## Results

### Normal Development and Morphology of the *Usp21*-deficient mice

**Fig 1:**
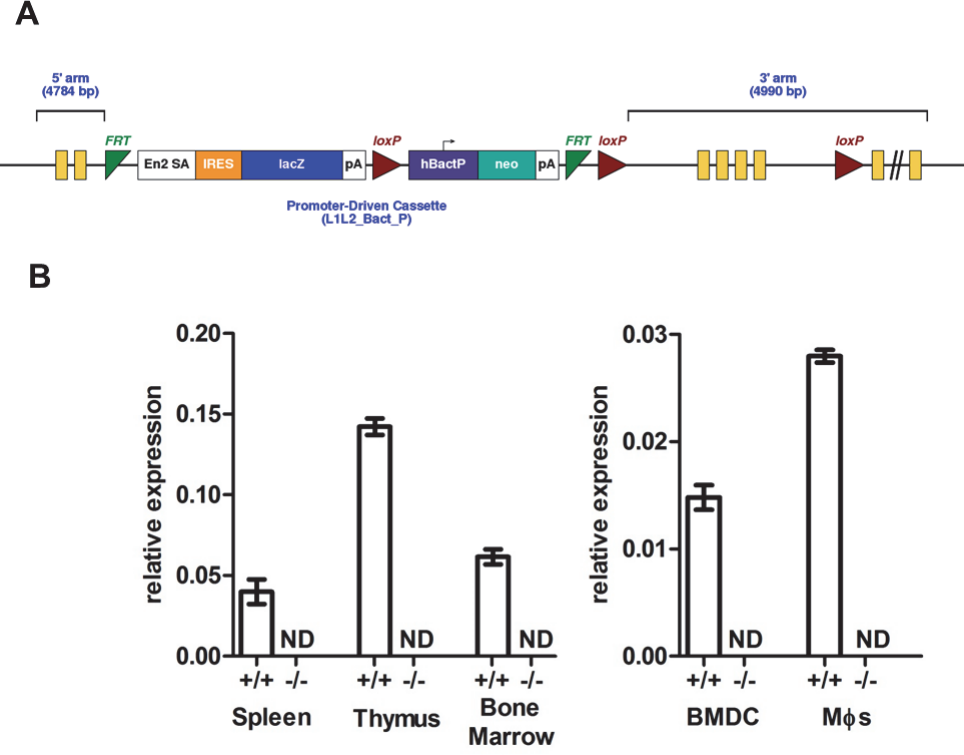
*Usp21*-knockout mouse line. (A) The structure of the *Usp21* ^tm1a(EUCOMM)Wtsi^ allele. (B) Relative expression of the *Usp21*-coding transcript (ENSMUST00000065941) in the spleen, thymus and bone marrow, as well as in the bone marrow derived dendritic cells (BMDCs) and macrophages (Mφ) of wild type and *Usp21* ^-/-^ mice. Analysis by qRT-PCR with *Usp21* expression for each sample normalizes to β-actin; bars represent mean ± SEM; data are from 4–7 mice per group, acquired in 3 independent experiments; ND – not detected.

The mouse strain carrying the *Usp21* ^tm1a(EUCOMM)Wtsi^ allele on the C57BL/6 background was derived from the *Usp21*-gene targeted ES-cell line EPD0716_10_H09, generated by the International Mouse Knockout Consortium [33,34,35]. The *Usp21* ^tm1a(EUCOMM)Wtsi^ allele carries a gene-trap DNA-cassette, inserted into the second intron of the *Usp21* gene, consisting of a splice acceptor site, an internal ribosome entry site (IRES) and a *β*-galactosidase reporter, followed by a neomycin resistance marker expressed from an independent β-actin promoter (Fig. 1A) [34]. The correct structure of the targeted allele was validated as outlined in the Materials and Methods. The use of the splice acceptor site in the cassette is predicted to generate a truncated transcript encoding the first 200 out of 566 amino acids of the USP21 protein, excluding any functional domains.

qRT-PCR analysis of the *Usp21* ^tm1a/tm1a^ mouse tissues confirmed that the *Usp21* ^tm1a(EUCOMM)Wtsi^ allele disrupted the production of *Usp21*-coding transcript ENSMUST00000065941. While qPCR-product corresponding to the junction of exons 2–3 was amplified from wild type mouse bone marrow, thymus and spleen, its levels were below the limit of detection in the tissues of *Usp21* ^tm1a/tm1a^ mice (Fig. 1B). The *Usp21* transcript was also not detectable in bone marrow derived dendritic cells (BMDCs) or macrophages of *Usp21* ^tm1a/tm1a^ mice (Fig. 1B). The mouse line is therefore referred to as *Usp21*-knockout (*Usp21* ^-/-^) in subsequent sections of this paper. Further analysis indicated some increase in the transcript levels of Usp21 homologues *Usp1* and *Usp12* in the bone marrow of *Usp21* ^-/-^ mice (S1 Fig.), suggesting that there may be some compensation by these proteins for the loss of Usp21 in certain cell types.

The *Usp21* ^-/-^ animals were viable, with no dysmorphology, and bred normally. There was no significant increase in the levels of embryonic mortality, in contrast to the previously characterized *Gata3* and *Ripk1* knockout mouse lines that exhibit either embryonic or perinatal lethality [13,26].

### Normal Hematopoietic Stem Cell Function in the *Usp21*-knockout mice

**Fig 2:**
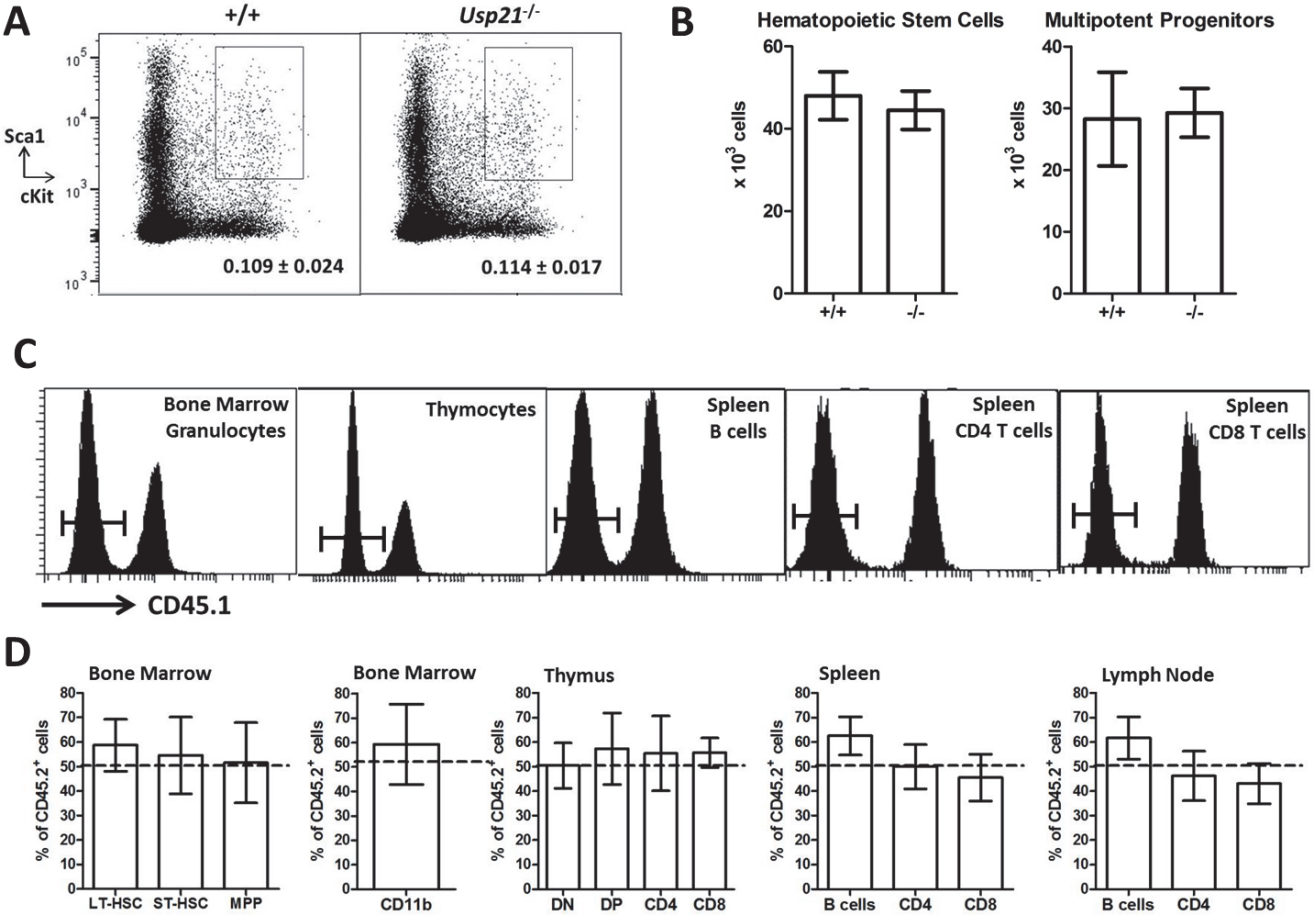
Normal Hematopoietic Stem Cell Numbers and Function in the *Usp21*-deficient mice. (A) Flow cytometry profiles of the mouse bone marrow, stained for lineage markers, cKit and Sca1, and gated on the lineage negative cells (CD11b^-^B220^-^TER119^-^CD4-CD8^-^). Lin^-^cKit^+^Sca1^+^ (LKS) population that contains hematopoietic stem cells and multipotent progenitors is highlighted. (B) Absolute numbers of hematopoietic stem cells (HSCs Lin^-^cKit^+^Sca1^+^Flt3^-^) and multipotent progenitors (MPPs, Lin^-^cKit^+^Sca1^+^Flt3^+^). Bars represent mean ± SEM, the presented data is from 4 mice per group, and is representative of 3 independent experiments with 14 mice per group analyzed in total; differences between wild type (+/+) and *Usp21*-knockout (-/-) mice are not statistically significant. (C-D) Competitive bone marrow chimeras: lethally irradiated mice transplanted with a 50:50 mix of *Usp21* ^-/-^ (CD45.2^+^) and wild type (CD45.1^+^) bone marrow cells, and analyzed at 20 weeks after the reconstitution. (C) Representative histograms showing the relative contribution of *Usp21* ^-/-^ (CD45.2^+^, left-peaks) and wild type (CD45.1^+^, right-peak) HSCs to the different hematopoietic lineages in the chimeras; and (D) data quantification. Bars represent means ± SEM, from 5 mice per group. Long-term HSCs are gated as Lin^-^cKit^+^Sca1^+^Flt3^-^CD34^-^, short-term HSCs as Lin^-^cKit^+^Sca1^+^Flt3^-^CD34^+^, and MPPs as Lin^-^cKit^+^Sca1^+^Flt3^+^CD34^+^; DN—double negative thymocytes (CD4^-^CD8^-^), DP—double positive thymocytes (CD4^+^CD8^+^). The contribution of *Usp21* ^-/-^ cells to all the lineages is not significantly different from 50%, indicating no defect in *Usp21* ^-/-^ stem cell function in direct competition with wild type cells.

GATA3 was previously shown to be essential for the maintenance of hematopoietic stem cells and for all stages of T cell differentiation in the thymus in mouse models [14,15,16,17]. In order to further explore possible functional interactions between USP21 and GATA3, we carried out an in depth characterization of hematopoiesis and lymphocyte differentiation in the *Usp21* ^-/-^ mice. The analysis was performed in both young (8–12 weeks) and aged (≈1 year) *Usp21* ^-/-^ mice against age-matched wild type controls (Table 1, S3 Table). The numbers of hematopoietic stem cells (HSCs) were normal in both young and aged *Usp21* ^-/-^ mice, as measured using either Lin^-^cKit^+^Sca1^+^Flt3^-^CD34^-^ or Lin^-^cKit^+^Sca1^+^CD48^-^CD150^+^ stem cell marker panels [36]; (Fig. 2A-B, and data not shown). The numbers of the major lymphoid, erythroid and myeloid cell types were also normal in young *Usp21* ^-/-^ mice (Table 1), further suggesting preservation of HSC function and normal progression of hematopoiesis. To assess the function of *Usp21* ^-/-^ HSCs in direct competition with wild type stem cells, mixed bone marrow chimeras were set up by reconstituting lethally-irradiated recipient mice with a 50:50 mix of *Usp21* ^-/-^ and wild type bone marrow. Analysis of the recipient mice at 20 weeks after the reconstitution showed normal contribution of *Usp21* ^-/-^ HSCs to the major hematopoietic lineages, as well as normal self-renewal capacity as shown by the reconstitution of the stem cell compartment in the recipient mice (Fig. 2C-D). This suggests that, in contrast to GATA3 [14,15], USP21 is not required for the normal maintenance of hematopoietic stem cell functions.

**Table 1:** Normal numbers of hematopoietic and immune cells in *Usp21* ^-/-^ mice.

Tissue and Cell Type	Flow Cytometry Gating	Cell Count Mean ± SD / x10^6^ cells		Mann-Whitney test
		Wild Type	*Usp21* ^-/-^	
**Bone Marrow**				
Hematopoietic Stem Cells	Lineage^-^ cKit^+^Sca1^+^Flt3^-^	0.048 ± 0.010	0.045 ± 0.008	p = 1.0, ns
Multipotent Progenitors	Lineage^-^ cKit^+^Sca1^+^Flt3^+^	0.028 ± 0.013	0.029 ± 0.007	p = 0.38, ns
Pro- and pre-B cells	B220^+^IgM^-^IgD^-^	3.5 ± 1.1	5.3 ± 1.1	p = 0.15, ns
Immature B cells	B220^+^IgM^+^IgD^-^	1.6 ± 0.5	2.1 ± 0.4	p = 0.56, ns
Mature B cells	B220^+^IgM^+^IgD^+^	1.3 ± 0.3	1.3 ± 0.1	p = 0.88, ns
Erythroblasts	CD71^+^TER119^+^	23.4 ± 2.4	21.5 ± 3.9	p = 0.69, ns
Erythrocytes	CD71^-^TER119^+^	20.5 ± 3.9	19.0 ± 4.4	p = 0.89, ns
**Thymus**				
Double negative cells	CD4^-^CD8^-^	11.0 ± 6.3	9.5 ± 4.1	p = 0.74, ns
Double positive cells	CD4^+^CD8^+^	235 ± 131	223 ± 70	p = 0.81, ns
CD4 single-positive cells	CD4^+^CD8^-^	19.1 ± 8.6	14.7 ± 5.2	p = 0.32, ns
CD8 single-positive cells	CD8^+^CD4^-^	6.1 ± 2.2	5.1 ± 1.7	p = 0.37, ns
**Spleen**				
Transitional B cells	B220^+^IgM^high^IgD^-^	11.6 ± 1.4	14.4 ± 3.7	p = 0.60, ns
Follicular B cells	B220^+^IgM^low^IgD^+^	40.0 ± 5.4	43.2 ± 10.7	p = 0.84, ns
CD4 T cells	CD4^+^	16.6 ± 1.8	18.5 ± 4.6	p = 0.42, ns
CD8 T cells	CD8^+^	10.6 ± 2.3	11.7 ± 3.0	p = 0.61, ns

Data acquired by flow cytometry, and is representative of 2 independent experiments with 8 mice per group analyzed in total; bone marrow cell numbers are indicated per one tibia and femur.

**Fig 3:**
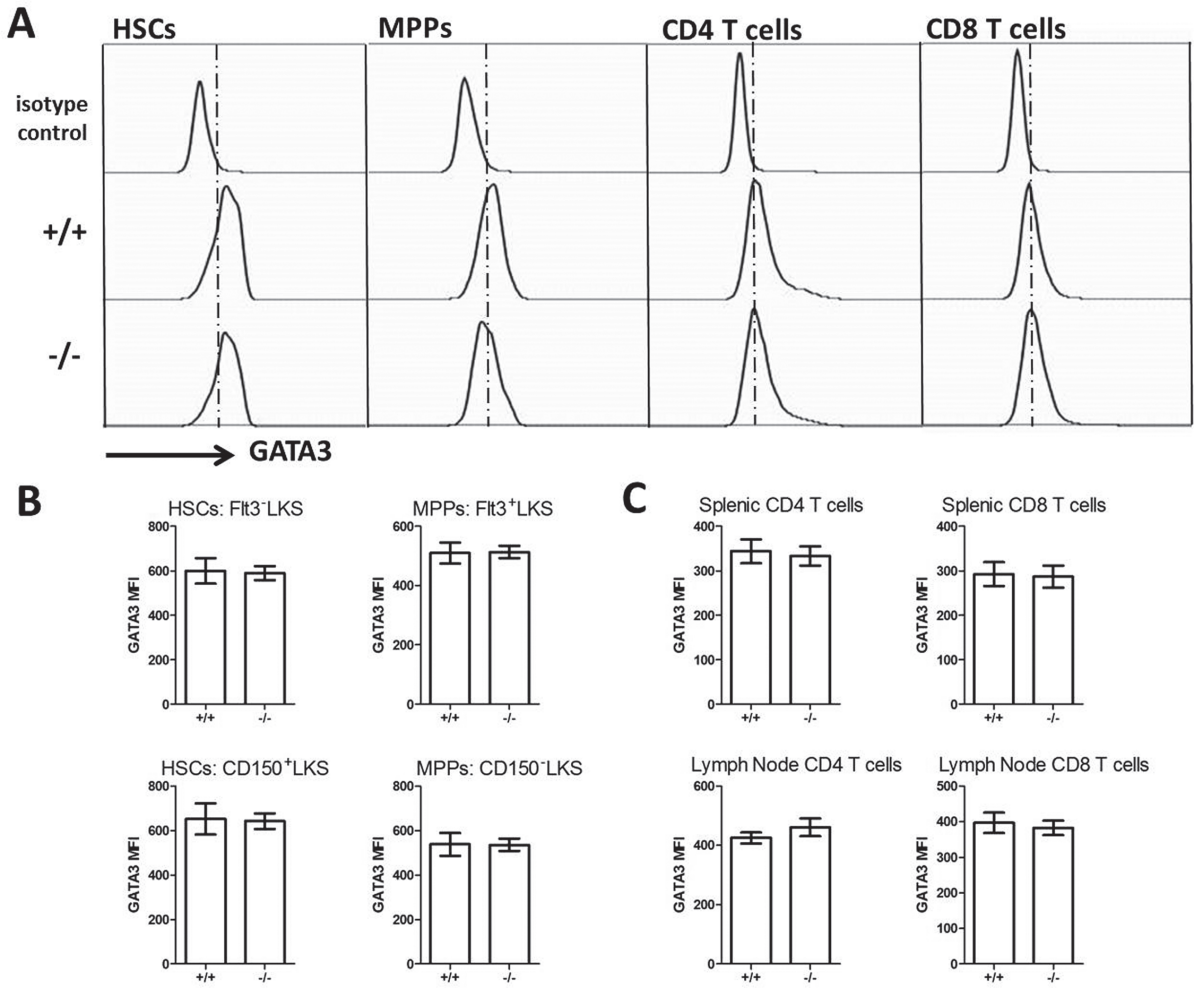
Intracellular Flow Cytometry Analysis of GATA3 levels in the *Usp21*-knockout mice. (A) Representative flow cytometry histograms of hematopoietic stem cells (HSCs, Lin^-^cKit^+^Sca1^+^Flt3^-^), multipotent progenitors (MPPs, Lin^-^cKit^+^Sca1^+^Flt3^+^), as well as CD4 and CD8 T cells from mouse spleen, stained with an isotype control antibody (top panel) or GATA3-specific antibody (bottom panels). (B) Mean fluorescence intensity (MFI) of GATA3-staining of HSCs (Lin^-^cKit^+^Sca1^+^Flt3^-^ or Lin^-^cKit^+^Sca1^+^CD150^+^), MPPs (Lin^-^cKit^+^Sca1^+^Flt3^+^ or Lin^-^cKit^+^Sca1^+^CD150^-^), and T cells from *Usp21* ^-/-^ and wild type mice. Bars represent mean ± SEM; data is from 10 mice per group, acquired in 2 independent experiments. There are no statistically significant differences in GATA3 MFI between the *Usp21* ^-/-^ and wild type cells.

To further explore the possible roles of USP21 in the regulation of GATA3 in the mouse bone marrow, we performed intracellular staining and flow cytometry analysis to measure GATA3 protein levels in *Usp21* ^-/-^ and control wild type hematopoietic stem and progenitor cells. No significant differences in the GATA3 levels were seen between the groups (Fig. 3A-B), indicating that USP21 does not play a major non-redundant role in the regulation of GATA3 protein levels in hematopoietic stem and progenitor cells.

### Normal T cell Development in the *Usp21*-knockout mice

**Fig 4:**
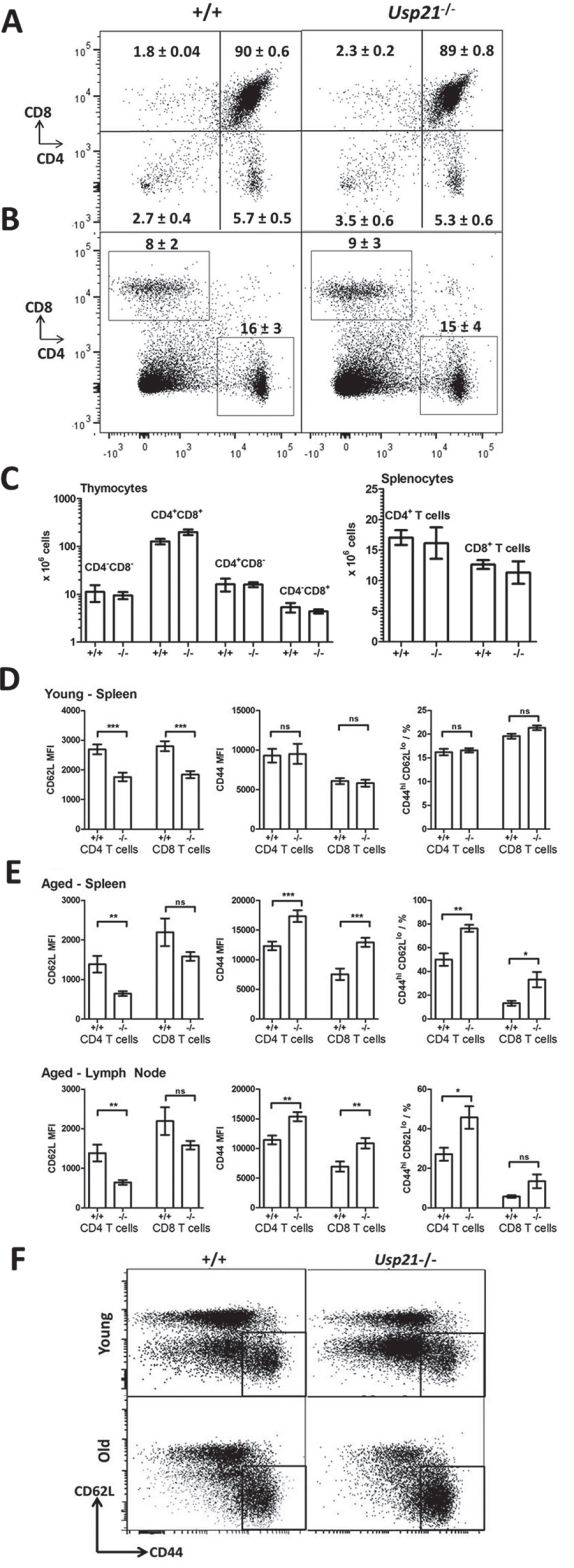
Normal T cell development and increased T cell activation in the *Usp21*-knockout mice. (A-B) Flow cytometry profiles of (A) thymus and (B) spleen of *Usp21* ^-/-^ and wild type mice, stained for CD4 and CD8. Average percentage of cells within the different gates is shown (mean ± SD). (C) Absolute numbers of thymocytes and splenic T cells in young *Usp21* ^-/-^ and wild type mice. (D-E) Expression of CD44 and CD62L activation markers on CD4 and CD8 T cells in the spleen and mesenteric lymph nodes of (D) young (8–12 weeks) and (E) aged (≈1year) mice. (C-E) Bars represent means ± SEM, data is from at least 10 mice per group, MFI—mean fluorescence intensity, statistical comparisons using ANOVA with Bonferroni multiple-comparisons post-hoc test; *** p<0.001, ** p<0.01, * p<0.05, ns-non-significant. (F) Representative flow cytometry plots showing CD62L and CD44 expression on splenic CD4 T cells in young and aged mice of *Usp21* ^-/-^ and wild type genotypes. The activated CD44^hi^CD62L^lo^ T cells gate is indicated.

T cell development in the *Usp21* ^-/-^ mice was analyzed in further detail, given the essential role of GATA3 in this process [16,17]. *Usp21* ^-/-^ mice had normal cellularity of the thymus and normal numbers of CD4 and CD8 T cells in the lymphoid organs (Table 1, Fig. 4 A-C), indicating that in contrast to GATA3, USP21 is dispensable for normal T cell development. Similarly, in the mixed bone marrow chimeras *Usp21* ^-/-^ cells contributed normally to T cells development, generating 53(±9)% of CD4 T cells and 48(±14)% of CD8 T cells (mean ± S.D.) in a direct 50:50 competition with wild type cells at 8 weeks after transplantation. Thymic T cell development was also maintained normally in aged *Usp21* ^-/-^ (≈1 year old, S3 Table), however there was a minor reduction in the numbers of splenic CD8 T cells in the aged *Usp21* ^-/-^ mice (S3 Table, p = 0.0495, n = 10) suggesting some disruption in peripheral T cell homeostasis in the aged *Usp21* ^-/-^ mice. We further observed some expansion of CD11c^+^MHCII^+^ dendritic cells and a trend towards B cells expansion in the spleens of the animals (S3 Table).

We further analyzed the expression of activation markers CD44, CD62L, and CD69 on CD4 and CD8 T cells, in both young (8–12 weeks) and aged mice (≈1 year old). Strikingly we observed enhanced T cell activation in the *Usp21*-knockout mice, affecting both CD4 and CD8 T cell subsets, and becoming progressively more severe with age (Fig. 4D-F). In young *Usp21*-knockouts, CD4 and CD8 T cells expressed lower levels of CD62L, indicating their more activated/antigen-experienced state, but the expression levels of CD44 or CD69 were not significantly different from those in the control group (Fig. 4D,F). As a result there was no significant increase in the percentage of T cells within the CD44^hi^CD62L^lo^ activated/antigen-experienced T cell gate. In contrast in the aged *Usp21*-knockout mice, there was an increase in CD44 and a reduction in CD62L levels on CD4 and CD8 T cells in spleen and mesenteric lymph nodes, resulting in a significant elevation in the proportion of CD44^hi^CD62L^lo^ activated/antigen-experienced T cells (Fig. 4E,F). Expression of co-stimulatory molecules and activation markers (CD80, CD86, MHCII) on antigen presenting cells (dendritic cells, macrophages or B cells) of aged *Usp21*-knockout mice was not significantly different from wild type (data not shown).

Intracellular flow cytometry was performed to measure GATA3 levels in CD4 and CD8 T cells of the aged *Usp21*-knockout mice, against age-matched wild type controls, however no differences in GATA3 levels were seen between the groups (Fig. 3A,C). In summary, the data indicates that in contrast to GATA3, USP21 is dispensable for normal thymic T cell differentiation. In peripheral lymphoid organs some dysregulation in T cell homeostasis was observed in *Usp21* ^-/-^ mice, with progressive increase in T cell activation with age, however this was not associated with alterations in GATA3 levels at least within the affected cells.

### Normal Responses to TLR and TNFR Stimulation

**Fig 5:**
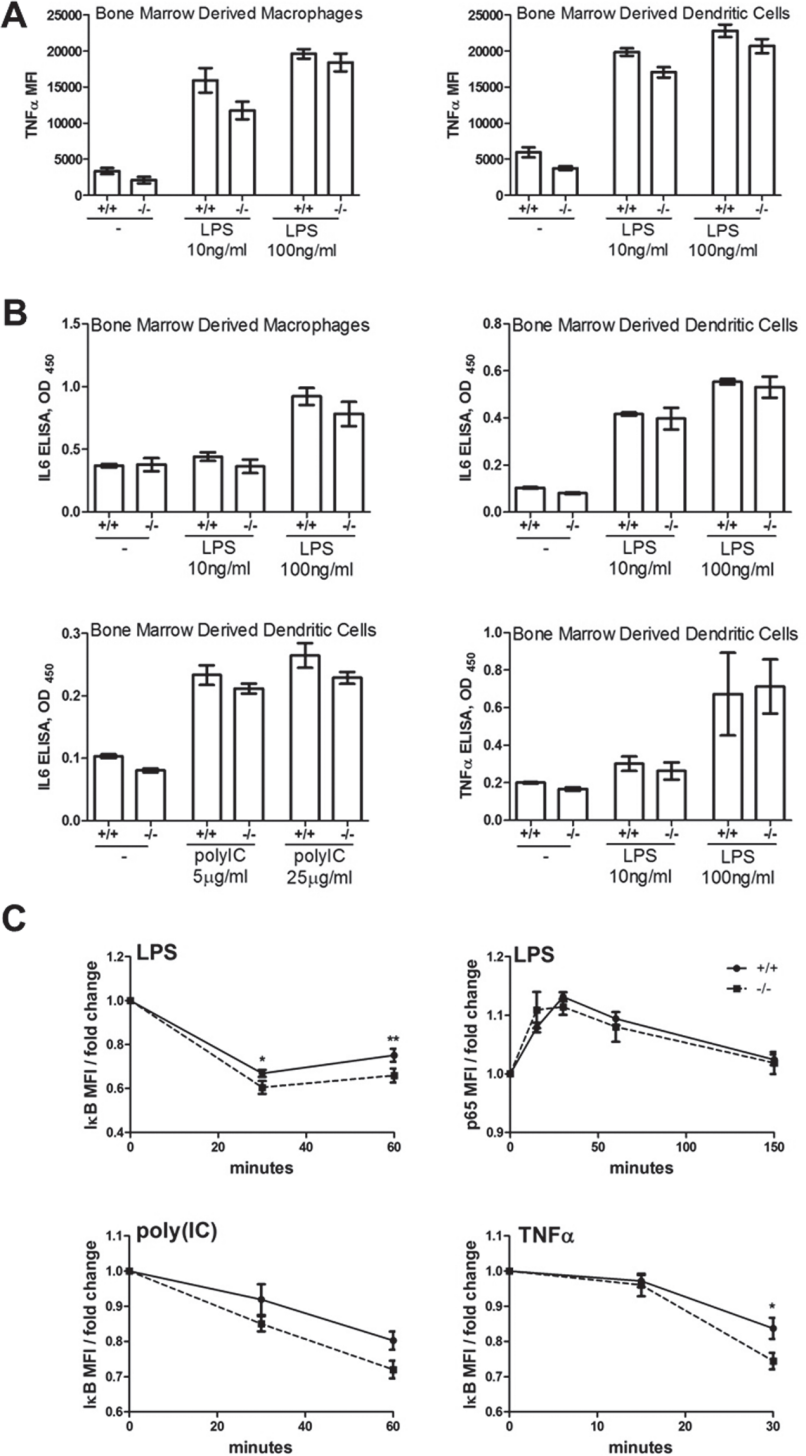
The responses of *Usp21* ^-/-^ (-/-) and control wild type (+/+) bone marrow derived macrophages and dendritic cells to TLR and TNFR stimulation. (A-B)The cells were stimulated over 18 hours with LPS at 10 and 100ng/ml, poly(IC) at 5 and 25 μg/ml, or TNFα at either 5 or 10ng/ml. (A) Production of cytokine TNFα was assessed by intracellular staining and flow cytometry, with the addition of Brefeldin A at 3μg/ml at 2 hours following the beginning of the stimulation. (B) Production of cytokines IL-6 and TNFα was assessed by ELISA. (C) Bone marrow derived macrophages from *Usp21* ^-/-^ (-/-, dashed line) and control wild type (+/+, solid line) mice were stimulated over 1.5 hours with LPS 100ng/ml, poly(IC) 25 μg/ml, or TNFα 10ng/ml, and analyzed by flow cytometry for IκBα levels and p65 NFκB S536 phosphorylation; data is presented as fold change in MFI over the untreated sample. Bars represent means ± SEM, datasets are from 4–5 mice per group with the cells from each mouse stimulated and analyzed in duplicate; MFI-mean fluorescence intensity; statistical analysis by ANOVA with Bonferroni post-hoc test, ** p<0.01, * p<0.05, and non-significant if no p-value is provided. All datasets were acquired however only datasets with a significant difference between stimulated and unstimulated samples are presented.

RIPK1 is a critical component of the signaling pathways linking Toll like receptors 3 and 4 (TLR3, TLR4) and TNFα-receptor 1 (TNFR1) to NFκB activation and inflammatory cytokine production. To explore the possible functional interactions between USP21 and RIPK1 in these pathways, we derived macrophages and dendritic cells from the bone marrow of *Usp21* ^-/-^ and wild types age-matched control mice, and compared their responses to TLR3, TLR4 and TNFR stimulation. The cells were stimulated with LPS (10 and 100ng/ml), poly(IC) at (5 and 25 μg/ml), and TNFα at (5 or 10ng/ml), and analyzed for expression of co-stimulatory molecules (CD80 and CD86) and production of inflammatory cytokines (TNFα and IL-6) by flow cytometry or ELISA (Fig. 5A-B, S2 Fig. all datasets were acquired however only datasets with a significant upregulation of activation markers or cytokine secretion above the level seen in unstimulated cells are presented). Additionally flow cytometry was used to measure IκBα degradation and p65 S536 phosphorylation, as a measure of NFκB signal transduction, in bone marrow derived macrophages (BMDMs) of *Usp21* ^-/-^ and wild types mice stimulated with in LPS, poly(IC), and TNFα (Fig. 5C). Mild increase in IκBα degradation but no differences in p65 phosphorylation were seen in the *Usp21* ^-/-^ cells (Fig. 5C). Furthermore, no significant changes in the biological responses of *Usp21* ^-/-^ cells, such as activation marker expression or inflammatory cytokine production, were observed (Fig. 5A-B). Further stimulations were performed with primary cells from the spleen and bone marrow of *Usp21* ^-/-^ and wild type mice, measuring co-stimulatory marker induction on CD11b^+^ total myeloid lineage cells or CD11c^+^ dendritic cell, however as previously, there were no significant differences in the responses of *Usp21* ^-/-^ and wild type cells (S3 Fig.).

### Normal Immune Response and Resistance to Bacterial Infectious Challenge in *Usp21*-knockout mice

**Fig 6:**
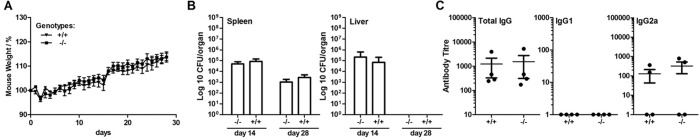
Normal resistance of *Usp21* ^-/-^ mice to *Salmonella typhimurium* infection. (A) Percent change in mouse body weight over the 28 day time-course of infection for *Usp21* ^-/-^ mice (-/-) and wild type control mice (+/+). (B) Bacterial counts (Log10 CFU/organ) at days 14 and 28 post-infection in spleen and liver. (C) Anti-TetC serum antibody titres at day 28 of infection, including total IgG, IgG1, and IgG2a isotypes. Bars show (A, C) mean ± SEM or (B) geometric mean ± SD.

In order to further explore immune system function in the *Usp21* ^-/-^ mouse line, the mice were challenged intravenously with S*almonella typhimurium*. The mice were weighed daily to monitor the progress of infection; spleens and livers were collected for the measurement of bacterial load at days 14 and 28, and serum was collected on day 28 to measure antigen specific antibody titres. There was no significant difference between wild type and *Usp21* ^-/-^ mice in any of the parameters measured, including body weight, tissue bacterial load, or antibody titres (Fig. 6). This suggests that loss of USP21 did not have significant impact on the immune resistance to bacterial challenge at least in this mouse infection model.

## Discussion

In this study we characterized a *Usp21*-deficient mouse model, and report that USP21 is dispensable for mouse embryonic development, hematopoietic stem cell functions or lymphocyte differentiation. The *Usp21*-knockout mice were born in normal Mendelian numbers with no obvious dysmorphology, and had normal hematopoietic stem cell functions and lymphocyte development. Furthermore, the biological responses of *Usp21*-deficient macrophages and dendritic cells to TLR and TNFR stimulation were not significantly altered, and the *Usp21*-knockout mice mounted effective immune response to bacterial challenge with *Salmonella typhimurium*. Given the essential role of GATA3 for normal mouse development, hematopoietic stem cell function and T lymphocyte differentiation [14,15,16,17], the data suggest that USP21 is redundant as a regulator of GATA3 activity in these settings. This is further validated by the normal GATA3 protein levels in hematopoietic stem cells, as well as CD4 and CD8 T cells of *Usp21*-knockout mice. Similarly, given the essential role of RIPK1 in the signaling pathways downstream of TLR3, TLR4, and TNFR [21,22,23], our data indicates that USP21 is unlikely to play a major biologically significant role in the regulation of RIPK1-dependent inflammatory responses in macrophages or dendritic cells.

Although our data indicates that USP21 is not an essential systemic regulator of GATA3 or RIPK1 activity during mouse development or hematopoiesis, it is important to emphasize that we cannot rule out that USP21 may regulate GATA3 or RIPK1 activities in some specialized cell types or under specific conditions that were not included in our study. Importantly, aged *Usp21*-knockout mice showed progressive increase in T cell activation in peripheral lymphoid organs, affecting both CD4 and CD8 lineages. The exact mechanisms underlying this phenotype are at present unclear and several hypotheses merit further investigation. Spontaneous T cell activation is also reported in the mouse line with a Treg-specific deletion of GATA3, and is associated with autoimmune pathology in aged mice [19]. Treg functions and GATA3-regulation within the Treg lineage of the *Usp21*-knockout mice will need to be addressed in future work. Alternatively, enhanced T cell activation may be linked to elevated type-I interferon response, as reported by Fan Y. *et al*. following viral challenge of the *Usp21*-knockout mice [11]. Given the driving role of type-I interferons in a range of autoimmune conditions [37,38], it will be important to address whether type-I interferon production is constitutively enhanced in naïve (uninfected) *Usp21*-knockout mice, and whether it can contribute to T cell activation and autoimmune pathology with age.

Analysis of the role of USP21 in GATA3 and RIPK1 regulation in other non-immune/hematopoietic cell types also merits further investigation. Our *Usp21*-knockout mouse line expresses a β-galactosidase reporter from the endogenous *Usp21*-promoter (Fig. 1A, and data not shown), thus allowing one to monitor *Usp21* promoter activity. Recently Clague et.al. reported that the *Usp21* transcript is most strongly expressed in the brain, reproductive and renal systems, based on consolidated data from EMBL gene atlas [2,39]. In the future this information may facilitate the identification of specific tissues, cell types, or conditions with highest *Usp21* expression, and thus enable the discovery of non-redundant physiological functions and substrates of USP21. Importantly, USP21 was previously shown to catalyze the deubiquitination of histone H2A and regulate gene expression in hepatocytes during liver regeneration [5]. This function of USP21 may now be further tested and explored in the knockout mouse model. In other studies USP21 was shown to localize to centrosomes and microtubules [8], and interact with MAP/microtubule affinity-regulating kinases 1–4 (MARKs) [7], however the biological significance of these interactions remains to be further addressed.

During the preparation of this paper a related study appeared online [11], identifying RIG-I as a novel substrate of USP21 using an independently generated *Usp21*-knockout mouse, and demonstrating the role of USP21 as a negative regulator of anti-viral immunity. Our work complements the findings of Fan *et.al*., addressing the role of USP21 in hematopoiesis, lymphocyte development, and immune response to bacterial infections. Overall the two papers provide a more complete picture of the roles of USP21 in hematopoiesis and immunity, and the two independently derived *Usp21*-deficient mouse lines will serve as valuable models for future characterization of the roles of USP21 in other aspects of mammalian physiology.

## Supporting Information

S1 Fig Expression levels of *Usp21* homologues and interacting partners in *Usp21* ^-/-^ mice. Relative expression of *Usp21*-homologues *Usp1, Usp2, Usp12, Usp43, Usp46* and USP21-binding partner *Mark2* in the spleen, thymus, bone marrow, and bone marrow derived dendritic cells (BMDC) of wild type and *Usp21* ^-/-^ mice. Analyses by qRT-PCR relative to β-actin; bars represent means ± SEM, data from 4 mice per group, statistical analysis using Mann-Whitney test in GraphPad Prism.
(TIF)

S2 Fig The responses of *Usp21* ^-/-^ (-/-) and control wild type (+/+) bone marrow derived macrophages and dendritic cells to TLR and TNFR stimulation. Bone marrow derived macrophages (A) and dendritic cells (B) were stimulated over 18 hours with LPS at 10 and 100ng/ml, poly(IC) at 5 and 25 μg/ml, or TNFα at 5 or 10ng/ml, and the expression of CD80 and CD86 activation markers analyzed by flow cytometry. Bars represent means ± SEM, datasets are from 4–5 mice per group with the cells from each mouse stimulated and analyzed in duplicate; MFI-mean fluorescence intensity; differences between the wild type and *Usp21* ^-/-^ are not statistically significant. All datasets were acquired, however only datasets with a significant difference between stimulated and unstimulated samples are presented.
(TIF)

S3 Fig The responses of *Usp21* ^-/-^ (-/-) and control wild type (+/+) antigen presenting cells from mouse spleen and bone marrow to TLR and TNFR stimulation. (A-B) Splenocytes and (C) bone marrow cells were stimulated over 18 hours with LPS at 10 and 100ng/ml, poly(IC) at 5 and 25 μg/ml, or mouse recombinant TNFα at 10ng/ml. Cell-surface expression of activation markers CD80 and CD86 was assessed by flow cytometry, gating on CD11b^+^ total myeloid lineage cells or CD11c^+^ dendritic cells. Bars represent means ± SEM, datasets are from 4 mice per group with the cells from each mouse stimulated and analyzed in duplicate; MFI – mean fluorescence intensity; differences between the wild type and *Usp21* ^-/-^ are not statistically significant. All datasets were acquired however only datasets with a significant difference between stimulated and unstimulated samples are presented.
(TIF)

S1 Table qRT-PCR Primer Sequences. (DOCX)

S2 Table Flow Cytometry Antibodies. Antibodies against the cell surface markers of different immune and hematopoietic cell types used in the flow cytometry analyses.
(DOCX)

S3 Table Numbers of hematopoietic and immune cells in aged *Usp21* ^-/-^ mice. Data acquired by flow cytometry, numbers presented are from 10 mice per group acquired in two independent experiments; bone marrow cell numbers are indicated per one tibia and femur; mouse age ≈ 1 year.
(DOCX)
