# Supplementary figures and images for: Ubiquitin Specific Protease 21 Is Dispensable for Normal Development, Hematopoiesis and Lymphocyte Differentiation
Source: PLoS One. 2015 Feb 13;10(2):e0117304. doi: 10.1371/journal.pone.0117304 (PMC4332479; doi:10.1371/journal.pone.0117304)

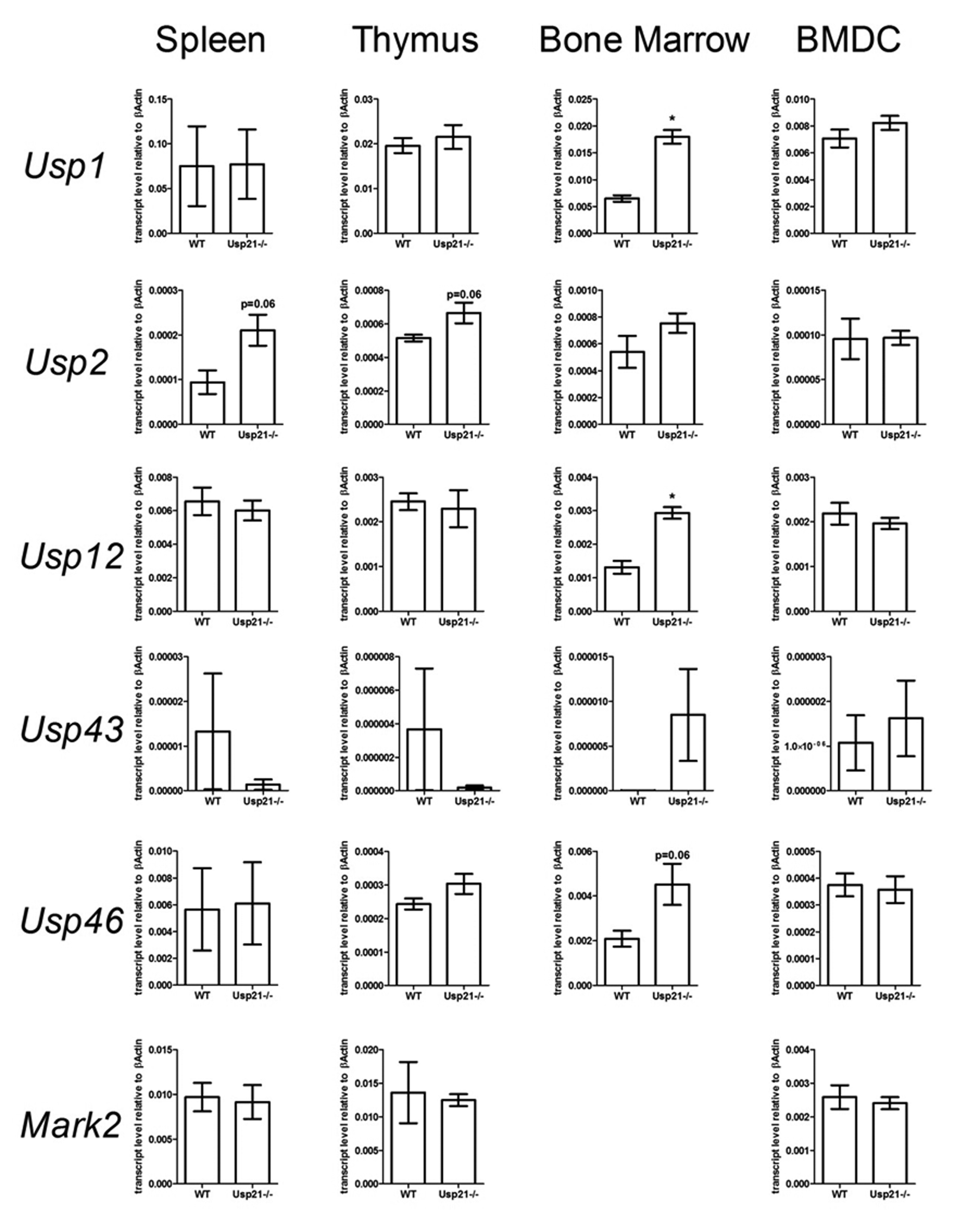

Supplement: S1 Fig — Relative expression of Usp21-homologues Usp1, Usp2, Usp12, Usp43, Usp46 and USP21-binding partner Mark2 in the spleen, thymus, bone marrow, and bone marrow derived dendritic cells (BMDC) of wild type and Usp21 -/- mice. Analyses by qRT-PCR relative to β-actin; bars represent means ± SEM, data from 4 mice per group, statistical analysis using Mann-Whitney test in GraphPad Prism. (TIF) [file pone.0117304.s001.tif]

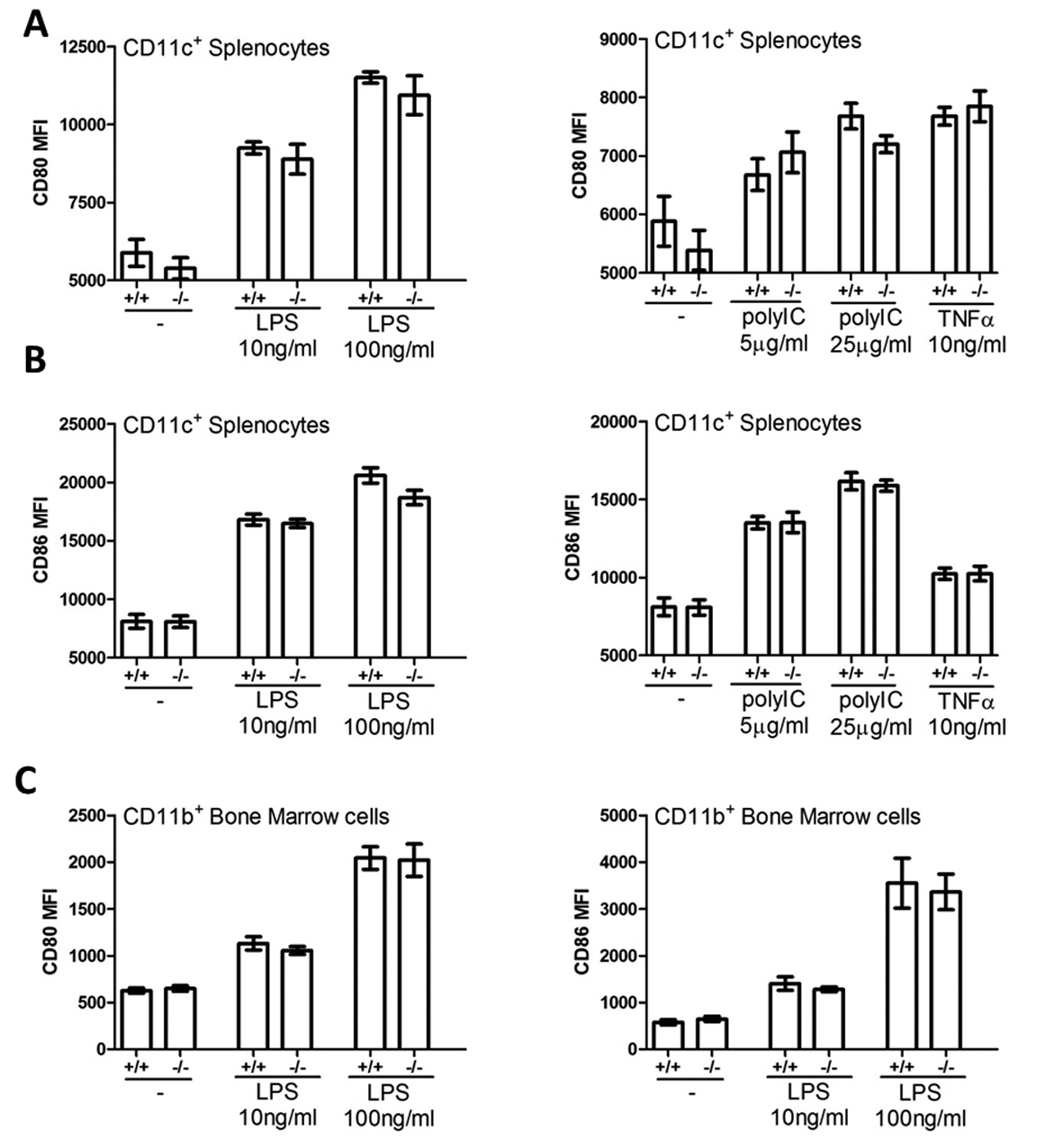

Supplement: S3 Fig — (A-B) Splenocytes and (C) bone marrow cells were stimulated over 18 hours with LPS at 10 and 100ng/ml, poly(IC) at 5 and 25 μg/ml, or mouse recombinant TNFα at 10ng/ml. Cell-surface expression of activation markers CD80 and CD86 was assessed by flow cytometry, gating on CD11b+ total myeloid lineage cells or CD11c+ dendritic cells. Bars represent means ± SEM, datasets are from 4 mice per group with the cells from each mouse stimulated and analyzed in duplicate; MFI – mean fluorescence intensity; differences between the wild type and Usp21 -/- are not statistically significant. All datasets were acquired however only datasets with a significant difference between stimulated and unstimulated samples are presented. (TIF) [file pone.0117304.s003.tif]
