# Supplementary material for: Ubiquitin Specific Protease 21 Is Dispensable for Normal Development, Hematopoiesis and Lymphocyte Differentiation
Source: PLoS One. 2015 Feb 13;10(2):e0117304. doi: 10.1371/journal.pone.0117304 (PMC4332479; doi:10.1371/journal.pone.0117304)
Supplement: S1 Table — (DOCX) [file pone.0117304.s004.docx]

| **Target and Primer Name** | **Primer Sequence (5’-3’)** |
| --- | --- |
| β-Actin_Fw | CTA AGG CCA ACC GTG AAA AG |
| β-Actin_Fw | ACC AGA GGC ATA CAG GGA CA |
| Mark2_Fw | TGT GAA GAT CAT CGA CAA GAC C |
| Mark2_Rv | CTT CAT TAT TCT TAC TTC TCG GAA CAG |
| Hprt_Fw | CAG GCC AGA CTT TGT TGG AT |
| Hprt_Rv | TTG CGC TCA TCT TAG GCT TT |
| Usp1_Fw | GAG CGA AGT CTC CTG TTT GAT |
| Usp1_Rv | GAA AGT CCA CCA CCG TAA CA |
| Usp2_Fw | CTA AGA GAC CTG GAC TTG AGA GA |
| Usp2_Rv | GAG TGA TTG GAC ACA GCA TAC A |
| Usp12_Fw | CTG TTC AAC ACG TCA GGA GAT G |
| Usp12_Rv | CAA TAT AAT GGC CTC GGT TGG G |
| Usp43_Fw | CTA TTC TAC CAG AAG CGG AAC AG |
| Usp43_Rv | CCA ATG ATC AGA CAA GGA GGA G |
| Usp46_Fw | CAT TGC CAC GCA GAA GAA GAA G |
| Usp46_Rv | GCA TCC TGC TGC ATG TAG TTA TC |
