# Supplementary material for: Ubiquitin Specific Protease 21 Is Dispensable for Normal Development, Hematopoiesis and Lymphocyte Differentiation
Source: PLoS One. 2015 Feb 13;10(2):e0117304. doi: 10.1371/journal.pone.0117304 (PMC4332479; doi:10.1371/journal.pone.0117304)
Supplement: S2 Table — Antibodies against the cell surface markers of different immune and hematopoietic cell types used in the flow cytometry analyses. (DOCX) [file pone.0117304.s005.docx]

| **Target** | **Fluorophore** | **Clone** | **Supplier** |
| --- | --- | --- | --- |
| CD23 | FITC | B3B4 | eBioscience |
| CD34 | FITC | RAM34 | eBioscience |
| CD48 | FITC | HM48-1 | eBioscience |
| CD62L | FITC | MEL-14 | Tonbo Biosciences |
| CD69 | FITC | H1.2F3 | eBioscience |
| CD11c | Alexa Fluor 488 | N418 | eBioscience |
| F4/80 | Alexa Fluor 488 | BM8 | BioLegend |
| CD80 | PE | 16-10A1 | eBioscience |
| CD135/FLT3 | PE | A2F10 | BioLegend |
| CD150 | PE | mShad | eBioscience |
| IgM | PE | II/41 | eBioscience |
| CD4 | PerCP/Cy5.5 | RM4-5 | BioLegend |
| CD8a | PerCP/Cy5.5 | 53-6.7 | BioLegend |
| CD11b | PerCP/Cy5.5 | M1/70 | BioLegend |
| CD45R/B220 | PerCP/Cy5.5 | RA3-6B2 | BioLegend |
| GR1 | PerCP/Cy5.5 | RB6-8C5 | eBioscience |
| F4/80 | PerCP/Cy5.5 | BM8 | eBioscience |
| TER119 | PerCP/Cy5.5 | TER119 | BioLegend |
| CD8a | APC | 53-6.7 | eBioscience |
| CD44 | APC | IM7 | eBioscience |
| CD86 | APC | GL1 | eBioscience |
| IgD | APC | 11-26c.2a | BioLegend |
| Sca1 | APC | E13-161.7 | BioLegend |
| Sca1 | APC | D7 | eBioscience |
| CD8a | Pacific Blue | 53-6.7 | BioLegend |
| CD11b | Pacific Blue | M1/70 | BioLegend |
| CD117/cKit | Pacific Blue | 2B8 | BioLegend |
| CD4 | PE-Cy7 | RM4-5 | BioLegend |
| CD11c | PE-Cy7 | N418 | BioLegend |
| CD19 | PE-Cy7 | 6D5 | BioLegend |
| CD21 | PE-Cy7 | eBio8D9 | eBioscience |
| CD71 | PE-Cy7 | RI7217 | BioLegend |
| CD117/cKit | PE-Cy7 | 2B8 | BD Biosciences |
| CD45 | APC-Cy7 | 30-F11 | BioLegend |
| CD45.1 | APC-Cy7 | A20 | BioLegend |
| I-A/I-E MHC class II | APC-Cy7 | M5/114.15.2 | BioLegend |
