# Supplementary material for: Ubiquitin Specific Protease 21 Is Dispensable for Normal Development, Hematopoiesis and Lymphocyte Differentiation
Source: PLoS One. 2015 Feb 13;10(2):e0117304. doi: 10.1371/journal.pone.0117304 (PMC4332479; doi:10.1371/journal.pone.0117304)
Supplement: S3 Table — Data acquired by flow cytometry, numbers presented are from 10 mice per group acquired in two independent experiments; bone marrow cell numbers are indicated per one tibia and femur; mouse age ≈ 1 year. (DOCX) [file pone.0117304.s006.docx]

| **Tissue and Cell Type** | **Flow Cytometry Gating** | **Cell Count Mean ± SD** **/ x10^6^ cells** | | **Mann-Whitney test** |
| --- | --- | --- | --- | --- |
|  |  | **Wild Type** | ***Usp21* ^-/-^** |  |
| **Bone Marrow** | | | | |
| Hematopoietic Stem Cells | Lineage^-^ cKit^+^Sca1^+^Flt3^-^ | 0.11 ± 0.06 | 0.14 ± 0.04 | p=0.31,ns |
| Multipotent Progenitors | Lineage^-^ cKit^+^Sca1^+^Flt3^+^ | 0.045 ± 0.015 | 0.055 ± 0.024 | p=0.32, ns |
| B cell lineage | B220^+^ | 12.1 ± 4.9 | 13.8 ± 4.5 | p=0.27, ns |
| Pro- and pre-B cells | B220^+^IgM^-^IgD^-^ | 8.3 ± 4.0 | 9.8 ± 3.3 | p=0.27, ns |
| Immature B cells | B220^+^IgM^+^IgD^-^ | 1.5 ± 0.6 | 2.0 ± 0.6 | p=0.10, ns |
| Mature B cells | B220^+^IgM^+^IgD^+^ | 2.2 ± 0.9 | 2.0 ± 1.6 | p=0.31, ns |
| Monocytes & Granulocytes | CD11b^+^ | 15.8 ± 3.6 | 16.0 ± 5.8 | p=0.68, ns |
| **Thymus** | | | | |
| Double negative cells | CD4^-^CD8^-^ | 5.7 ± 2.4 | 5.4 ± 1.3 | p=1.0, ns |
| Double positive cells | CD4^+^CD8^+^ | 67.5 ± 29.3 | 56.6 ± 9.9 | p=0.84, ns |
| CD4 single-positive cells | CD4^+^CD8^-^ | 8.4 ± 3.8 | 7.9 ± 0.8 | p=0.68, ns |
| CD8 single-positive cells | CD8^+^CD4^-^ | 2.8 ± 1.5 | 2.5 ± 0.6 | p=1.0, ns |
| **Spleen** | | | | |
| Transitional B cells | B220^+^CD21^lo^CD23^lo^ | 5.6 ± 0.9 | 11.1 ± 7.0 | p=0.10,ns |
| Follicular B cells | B220^+^CD21^mid^CD23^mid^ | 36.8 ± 10.1 | 54.9 ± 14.7 | p=0.10, ns |
| Marginal Zone B cells | B220^+^CD21^hi^CD23^lo^ | 4.4 ± 1.9 | 5.9 ± 3.2 | p=0.53, ns |
| CD4 T cells | CD4^+^ | 18.0 ± 2.9 | 17.3 ± 2.7 | p=0.84, ns |
| CD8 T cells | CD8^+^ | 10.5 ± 2.4 | 5.6 ± 2.2 | p=0.05 (*) |
| Granulocytes | CD11b^+^GR1^+^ | 1.5 ± 1.2 | 3.2 ± 2.9 | p= 0.44 |
| Dendritic Cells | CD11c^+^MHCII^+^ | - 1. ± 0.7 | 2.0 ± 1.5 | p=0.02 (*) |
| Macrophages | CD11b^+^F4/80^+^MHCII^+^ | 3.7 ± 2.7 | 4.1 ± 2.5 | p=0.58 |
| **Mesenteric Lymph Nodes** | | | | |
| B cells | B220^+^ | 3.0 ± 0.8 | 8.0 ± 4.2 | p=0.11,ns |
| CD4 T cells | CD4^+^ | 4.0 ± 1.1 | 3.8 ± 1.4 | p=0.90, ns |
| CD8 T cells | CD8^+^ | 3.4 ± 1.0 | 2.6 ± 1.2 | p=0.46, ns |
